# Editorial: Imaging the immune response in inflammatory preclinical *in vivo* models

**DOI:** 10.3389/fimmu.2023.1197819

**Published:** 2023-04-24

**Authors:** M. Erreni, G. Vande Velde, R. Weigert, N. Devoogdt

**Affiliations:** ^1^ Unit of Advanced Optical Microscopy, IRCCS Humanitas Research Hospital, Milan, Italy; ^2^ Department of Biomedical Sciences, Humanitas University, Milan, Italy; ^3^ Biomedical MRI/MoSAIC, Department of Imaging and Pathology, Faculty of Medicine, KU Leuven, Leuven, Belgium; ^4^ Laboratory of Cellular and Molecular Biology, Center for Cancer Research, National Cancer Institute, National Institutes of Health, Bethesda, MD, United States; ^5^ Medical Imaging Department, *In vivo* Cellular and Molecular Imaging Laboratory, Vrije Universiteit Brussel, Brussels, Belgium

**Keywords:** imaging, inflammation, *in vivo* models, preclinical imaging, immune response

The immune response to pathological conditions is the result of a complex and coordinated activity of specialized cells, tissues and organs, aimed at promoting an effective host defense. Although *in vitro* models have been used to describe several aspects of the immune system, they often fail to reconstitute the dynamic complexity of *in vivo* multicellular systems. In the last decades, major breakthroughs in the fields of imaging and mouse engineering have opened the door to investigate such a complexity. Indeed, the possibility to select the appropriate imaging technique to address a specific biological question has made it possible to unveil novel aspects of the immune response at different levels of temporal and spatial resolution.

The aim of this Research Topic is to provide an overview of the different imaging technologies and their application to analyse immune responses in *in vivo* models.

Conventional histology and immunohistochemistry represent the techniques of choice to visualize the expression of inflammatory mediators in pathological tissues. He et al. demonstrated, by immunohistochemistry, Hematoxylin & Eosin and Masson staining that Sinomenin, a biologically active alkaloid isolated from the roots and stems of the plant *Sinomenium acutum*, suppresses epithelial to mesenchymal transition in a mouse model of ovalbumin-induced asthma.

Besides *ex vivo* techniques, a variety of non-invasive imaging methodologies were developed to study the biodistribution and tissue accumulation of targeting probes and drugs in preclinical models of disease. Positron Emission Tomography (PET) and Single Photon Emission Computed Tomography (SPECT) are the most widely used, due to their capability to provide information about the metabolic and molecular activity of the analysed tissue. Moreover, PET and SPECT are routinely used in patients, thus facilitating the translation of data from pre-clinical models to human clinical practice. De Vlaminck et al. and Sandker et al. provided examples of SPECT to study the pharmacokinetics of targeting molecules. De Vlaminck et al. generated nanobodies able to recognize the signal regulatory protein alpha (SIRPα) for the SPECT targeting of tumor-associated myeloid cells in a mouse model of glioblastoma. They showed that the monovalent format of anti-SIRPα nanobodies has an improved tumor penetration compared to the bivalent counterpart and that myeloid cells infiltrated glioblastomas even in absence of blood-brain barrier permeabilization. Sandker et al. analyzed the effect of infectious stimuli in the pharmacokinetic and tumor targeting capability of an anti-PD-L1 antibody. They found that anti-PD-L1 antibody pharmacokinetics and tumor accumulation can be altered by a severe inflammatory response, thus requiring an appropriate modulation of the injected dose of the tracer.

Similarly, Rothlauf et al. applied PET and radiolabeled anti-CD8 nanobodies to track CD8^+^ T cells infiltration in a mouse model of influenza A virus infection. They visualized CD8^+^ T cells throughout the progression of the infection, showing that CD8^+^ T cells initially accumulate in mesenteric lymph nodes, then migrate into the infected lungs, and finally exit the lungs as the mice recovered. Likewise, using non-invasive optical imaging and PET, Traenkle et al. generated anti-human CD4 nanobodies to visualize and quantify CD4^+^ T cells in human tumor xenogratfs and in CD4^+^ T cell-rich tissue in human CD4 knock-in mice. Waaijer et al. used PET to study, for the first time, the biodistribution of a CSF1R-binding monoclonal antibody. They demonstrated that anti-CSF1R monoclonal antibodies do not exclusively target tumor macrophages but preferably distribute to other organs with high macrophage infiltration, highlighting the need for more studies to enhance the understanding of macrophage-specific antibodies for their potential application as targeting agents in humans. The use of CSF1R targeting strategies in the context of neurological disorders was also reviewed by Barca et al. They revised the supporting evidence on the role of CSF1R in neurological disorders and the efficacy of CSF1R-inhibition as therapeutic strategy. In addition, they discussed the recent development of *in vivo* molecular imaging of CSF1R, with a specific focus on the translocator protein (TSPO)-PET as a CSFR1 inhibition therapy readout.

Macrophages represent an interesting target not only in tumors but also in the onset of other inflammatory disease. Steinz et al. reviewed the application of folate-based PET for macrophage targeting in rheumatoid arthritis. Radiolabeled folate interacts with folate receptor β expressed on macrophages and it is a useful tracer in both preclinical and clinical applications. Similarly, Palani et al. showed that the glutamine analog (2S,4*R*)-4-^18^F-fluoroglutamine (^18^F-FGln) can be used for PET of macrophage metabolic activity in a mouse model of atherosclerosis. They experimentally showed that ^18^F-FGln PET is superior to ^18^F-FDG PET in detecting inflamed atherosclerotic lesions in mice, paving the road for its clinical application.

Although PET and SPECT are useful to study the biodistribution of injected probes and the metabolic activity of diseased tissues, they do not provide anatomic information on the organs of interest. For this reason, PET and SPECT are usually combined with computed tomography (CT) or magnetic resonance imaging (MRI), thus linking accurate spatial localization with metabolic/molecular data. Examples of this combined application were reported by De Vlaminck et al., Traenkle et al., Palani et al. and Sandker et al. Moreover, dedicated microCT can be also used as a stand-alone technique to monitor structural changes in tissues. Nagai et al. applied microCT to visualise the morphological and histological properties of peripheral and temporomandibular joints (TMJ) in a mouse model of rheumatoid arthritis with or without the exposure to mechanical strain on the TMJ. 3D morphological evaluation by microCT revealed morphological changes in the posterior part of the mandibular condyle and signs of bone destruction in a mouse model of anti-collagen antibody-induced arthritis.

As shown by Traenkle et al., beside PET and SPECT, optical imaging can be used to monitor biological process in live animals. However, a drawback of this technique is that light scattering and absorption by tissues limit its applicability for non-invasive imaging at high spatial resolution. Less subjective to these limitations are techniques such as fluorescence-guided surgery and intravital microscopy, that have been developed for the visualization of surgically exposed organs, allowing the possibility to analyse biological processes at both cellular and subcellular level. Pizzagalli et al. discussed the investigation of the migratory patterns of immune cells in living animals by intravital microscopy. They described the morpho-dynamic properties associated with immune cell migration during immune responses and the computational methods employed for their quantification. Moreover, recent advances in super-resolution microscopy have enabled the analysis of biological events in living organisms beyond the light diffraction limits. In this context, Johanson et al. reviewed the usefulness of this technique to investigate immune cell function and molecular processes underlying inflammatory responses and to answer long-standing fundamental questions in this matter.

In conclusion, a variety of imaging methodologies, each with their distinct features and possible applications, are available for researchers to investigate the complexity of the immune response in living animals. Differently from conventional *ex vivo* imaging methods, that generally provide snapshot data from fixed samples, *in vivo* imaging approaches, such as intravital microscopy and whole-body imaging techniques (PET, SPECT, CT, MRI, etc.), have the great potential to add the temporal dimension to immunological investigations. The choice of the right imaging technique, or even better, the combination of appropriate imaging methodologies strictly depends on the biological questions asked, aimed at collecting as much information as possible from single experiments in animal disease models.

## Author contributions

All authors have made a substantial, direct and intellectual contribution to the work. ME drafted the manuscript. The editors GVV, RW and ND provided critical revisions and approved the submitted manuscript.

